# Strategies in Infertile Azoospermic Patients with Negative Microdissection Testicular Sperm Extraction Surgery

**DOI:** 10.5152/tud.2020.20435

**Published:** 2023-03-01

**Authors:** Tharu Tharakan, Rong Luo, Daniel Foran, Miles Smith, Channa N Jayasena, Suks Minhas

**Affiliations:** 1 Department of Urology, Imperial Healthcare, NHS Trust, Charing Cross Hospital, London, UK; 2 Section of Investigative Medicine, Department of Medicine, Imperial College London, London, UK

**Keywords:** Andrology, azoospermia, male infertility

## Abstract

Non-obstructive azoospermia is reported to affect 1 in 100 men, and despite advances in surgical practice, the succesful sperm retrieval rate for microdissection testicular sperm extraction surgery (mTESE) is only 46%. This article reviews the potential causes for mTESE failure and provides a management strategy to guide the clinicians on how to treat this challenging cohort of patients.

**Main Points**

- *A recent meta-analysis reported that the sperm retrieval rate from mTESE was 46%.[7] Therefore, counseling a patient regarding a failed mTESE is not uncommon. Unfortunately, there is a paucity of well-designed large-scale studies to guide the clinician on the management strategies in this scenario.*
- *Surgical experience and embroylogical techniques can effect surgical sperm retrieval rates.*
- *Hormone stimulation, FNA mapping and Varicocele repair have all been advocated as methods to optimse sperm retrieval but there are insufficient data to support this and further propective randomised controlled trials are needed.*

## Introduction

The World Health Organization (2010) guidelines^1^ define azoospermia as the absence of sperm in the ejaculate. Azoospermia is estimated to be present in 1% of the population and 10%-20% of the patients presenting to an infertility clinic.^2^ Non-obstructive azoospermia (NOA) occurs when there is an impairment of spermatogenesis and has been reported to affect 1 in 100 men^2^ and accounts for 60% of all cases of azoospermia.^3^

Historically, couples with azoospermia were restricted to using either sperm donation or adoption. However, the development of surgical sperm retrieval coupled with assisted reproductive technologies (ART) resulted in the first child being conceived from a man with NOA in 1995.^4^

Conventional testicular sperm extraction (cTESE) involves random biopsies of the testicle. In 1999, Schlegel^5^ reported the technique of microdissection testicular sperm extraction (mTESE), which used optical magnification to identify the larger and opaque seminiferous tubules that are more likely to contain sperm. Meta-analyses^6,7^ have confirmed that mTESE has a comparable or higher surgical sperm retrieval rate than cTESE albeit, with a significant reduction in the testicular tissue removed.^5,8,9^ Although globally, mTESE has been adopted as the gold standard for surgical sperm retrieval, a recent meta-analysis highlighted that the overall success of mTESE was only 46%.^7^ However, the 6 largest mTESE studies in this metaanalysis (Table 1)^10-15^, showed -a significant discrepancy in the successful sperm retrieval rate, ranging from 22.1% to 56%. This review highlights the potential causes for mTESE failure and provides management strategies for this cohort of patients.

## Classification of Azoospermia

To understand the mechanisms that contribute to mTESE failure, one must first be aware of the different NOA histological subtypes. NOA is typically classified into 3 histological subclasses: hypospermatogenesis, maturation arrest, and Sertoli cell only (SCO).^16^ Hypospermatogenesis is characterized by the presence of spermatozoa of all stages of spermatogenesis although with significant reductions in quantity.^10^ Maturation arrest occurs when the germ cells fail to complete the maturation stage of spermatogenesis and is typically subdivided into early stage, where spermatogonia and spermatocytes are present, and late stage, where spermatids but no spermatozoa are detected.^17^ SCO is defined by the complete absence of germ cells.^10^ Patients with NOA commonly exhibit mixed histological patterns,^18^ but the predominant histological pattern has been reported to determine sperm retrieval rates.

Contemporary literature suggests that the successful sperm retrieval rate is the highest in hypospermatogenesis (73%-100%), followed by late maturation arrest (27%-86%) and early maturation arrest (27%40%).^19^ SCO is associated with poor surgical sperm retrieval rates (22.5%-41%).^19^ Therefore, although NOA histological subtypes have no value in the context of a primary mTESE, it may be useful in counseling the patients regarding the success of a secondary procedure.

## Procedural Factors

The causes for mTESE failure may be related to surgical and embryological factors.

## Surgical experience

There are several studies showing a learning curve for surgeons performing mTESE. Ishikawa et al.^20^ studied the outcomes for a single surgeon’s mTESE procedures. The authors subdivided the first 150 mTESE procedures into 3 chronological cohorts (first, middle, and last). There were no differences in the clinical or histopathological characteristics between the 3 groups. However, the authors observed that the successful sperm retrieval rate was significantly higher in the middle (44%) and last (48%) cohorts of patients than in the first 50 procedures (32%) (p<0.05). Moreover, in a sub-analysis of patients with SCO, a significantly higher sperm retrieval rate was observed in the middle and last cohort of patients than the first cohort (p<0.05). In addition, the operation time was significantly shorter in the middle (90±24 minutes) and the last groups (85±18 minutes) compared to the first group (114±32 minutes) (p<0.05). There were no postoperative complications in this series of patients. This study suggests that a minimum of 50 cases are needed for optimal mTESE outcomes. Similarly, Franceschelli et al.^21^ retrospectively analyzed the mTESE outcomes of an individual surgeon at a single institution. The mTESE procedures (n=122) were divided sequentially into 3 cohorts, and there was a significant increase in the sperm retrieval rate in consecutive years (p=0.01). The authors reported that there was a significant increase in the sperm retrieval rate after the surgeon’s first 50 cases.

Miyagawa et al.^22^ investigated mTESE outcomes in a single institution. The authors divided 200 consecutive patients who underwent mTESE chronologically into 4 equal cohorts. The patient groups were matched in age, testicular volume, testicular histology, and hormone profile. The authors reported that the operating time significantly decreased after the first 50 mTESE cases (p=0.0004). There was no statistically significant difference in the overall sperm retrieval rate between the cohorts, but multivariable logistic regression analysis revealed that the sperm retrieval rate for SCO increased significantly after the first 60 cases (p=0.0043).

Hsiao et al.^23^ retrospectively reviewed 1041 mTESE procedures over a 12-year period at a single institution and reported that although the overall successful sperm retrieval rate did not significantly change over the time period, there was an increase in the sperm retrieval rate for SCO (although this was not statistically significant) when stratifying by histology.

The mentioned studies suggest a learning curve for mTESE, especially in patients with SCO syndrome. However, in most studies, the threshold appears to be 50 cases; therefore, a further attempt at mTESE on the rationale of surgeon inexperience could only be justified using this threshold. Moreover, the literature is limited with only smallscale retrospective studies analyzing the learning curve of mTESE.

## Embryological factors

There are no studies analyzing the learning curve of embryologists for mTESE. However, there are data demonstrating that the embryological extraction process can affect the sperm retrieval rate. Crabbé et al.^24^ observed that in the testicular samples that had undergone conventional extraction methods (mincing and use of erythrocytelysing buffer) where no sperm was identified, the application of enzymatic digestion with collagenase type IV resulted in sperm retrieval in approximately 25% of cases. Other studies have reported that in cases where no spermatozoa were identified, use of enzymatic digestion with deoxyribonuclease and collagenase type IV yielded sperm in 9%^25^ and 25%^26^ of the patients.

This highlights the importance of testicular tissue processing by an embryologist in enhancing the sperm retrieval rate.

## Optimization

In cases of mTESE failure, hormone stimulation therapy has been advocated to optimize spermatogenesis, and fine-needle aspiration (FNA) mapping has been used to identify any focal areas of spermatogenesis before performing mTESE.

## FNA mapping

Testicular mapping involves FNA at predetermined sites of the testicle such that all the testicular tissue is systematically sampled. The subsequent histological analysis provides a geographical summary of where spermatogenesis is present in the testicle. This approach to surgical sperm retrieval has been advocated on the basis that it is less likely to miss any focal areas of spermatogenesis as it systematically samples all the areas of the testicle. Another advantage of FNA is that it prevents unnecessary biopsies of the testicle and thus reduces the risk of testicular atrophy and hypogonadism. However, critics of this technique argue that it does not retrieve the sperm and necessitates a further surgical sperm retrieval procedure, which potentially increases morbidity and is not cost-effective. Jarvis et al.^27^ reported that in a cohort of 82 men who had a previously failed mTESE, the use of FNA mapping identified at least 1 site of spermatogenesis in 29.3% (28/82). Furthermore, of those who were found to have spermatogenesis, 15 men underwent mTESE, and all had successful sperm retrieval. Therefore, FNA mapping could be used in those with failed mTESE to identify if there are any areas of focal spermatogenesis. However, the evidence for this treatment strategy is limited because of the paucity of controlled trials, and it could also be argued that without an appropriate control, the increase in surgical sperm retrieval rate observed with adjuvant FNA mapping may simply be a reflection of an expected increase in the cumulative success rates after repeated sperm retrieval attempts. Furthermore, it may be related to a different operating surgeon and expertise. Indeed, Dabaja et al.^9^ reported a successful sperm retrieval rate of 10% for mTESE in men with failed mTESE elsewhere. Talas et al.^28^ reported a retrospective analysis of 68 men who underwent mTESE. The authors reported a secondary successful sperm retrieval rate in 60% (3/5) of men.

## Hormone stimulation

The majority of men with NOA presenting with infertility will have hypergonadotropic hypogonadism or normal hormone status,^29^ and there is evidence that hormone stimulation therapy can improve surgical sperm retrieval rates and facilitate production of sperm in the ejaculate.^16,30^ The clinical justification for using hormone stimulation therapy is that it can potentially increase intratesticular testosterone (ITT), which is required for spermiogenesis. The ITT level has been reported to be significantly higher than serum testosterone level, with reports varying from 100 to 1000 times.^31^ However, the only method to measure ITT is using testicular aspiration, which is an invasive procedure, and thus hormone stimulation therapy has been utilized empirically.

Several clinical methods have been tested, including direct gonadotropin therapy, aromatase inhibitors, and selective estrogen receptor modulators (SERMs).

Gonadotropin therapy stimulates ITT,^32,33^ and human chorionic gonadotropin (hCG) and human menopausal gonadotropin (hMG) are imitations of luteinizing hormone and follicle-stimulating hormone (FSH), respectively.^34^

Aromatase inhibitors prevent the conversion of testosterone to estradiol in the Leydig cells of the testes, which reduces the negative feedback of estradiol on the hypothalamus-pituitary-gonadal axis. Men with infertility have been reported to have a low testosterone to estradiol ratio (T/E2).^35^ A T/E2 ratio of <10 has been described as the threshold for aromatase inhibitor therapy in men with NOA. Schiff et al.^36^ used testolactone or anastrazole (±hCG) in a cohort of men with NOA with Klinefelter’s syndrome before mTESE. The authors reported an overall successful sperm retrieval rate of 69%, and the sperm was retrieved in 6/6 (100%) of the anastrazole±hCG group and 21/32 (65%) in the testolactone±hCG group. There has also been a case report of a man with NOA with hypospermatogenesis who was treated with letrozole for 3 months^37^ and produced sperm in his ejaculate.

SERMs inhibit the estrogen receptors in the pituitary gland to block the negative feedback of estradiol, thus up-regulating gonadotropin secretion. Hussein et al.^38^ treated 492 men with NOA with clomiphene citrate±hCG or hCG+hMG. The authors observed that 54/492 (10.9%) men in the treatment group subsequently produced sperm in their ejaculate after hormone therapy, and 252/492 (51.2%, p<0.01) had a successful mTESE. However, it must be noted that 39/116 (33.9%) had a successful mTESE in the control group.

In the context of a failed mTESE, there have only been 4 studies that have used hormone stimulation, and all have used gonadotropin therapy. Shirashi et al.^16^ investigated the effects of gonadotropin therapy in men with NOA and primary hypogonadism who had failed mTESE previously. The treatment group were given hCG and also FSH, if their endogenous gonadotropin levels decreased. The control group included 20 men who received no hormone stimulation therapy and proceeded to secondary mTESE. The successful sperm retrieval rate was significantly higher in those receiving hormone stimulation therapy than the control group (21% vs 0%, p<0.05). Shiraishi et al.^39^ also reported a case series of 21 men with NOA and with hypergonadotrophic hypogonadism who were treated with hCG and FSH. All men had a failed mTESE, and only the men with hypospermatogenesis or late maturation arrest (n=2) were successful at the second mTESE after hormonal stimulation.

Selman et al.^40^ treated 49 men with NOA who had previously failed cTESE, with 4 months of recombinant FSH therapy, followed by 2 months of hCG. All the participants had normal hormone profiles and a histological diagnosis of maturation arrest. A repeat cTESE was performed after hormone stimulation therapy, and sperm was retrieved in 11/49 men, resulting in 3 full-term pregnancies. Hu et al.^32^ performed a case-control study in men with compensated hypergonadotrophic hypogonadism who had failed cTESE. In the treatment arm, 25 men received goserelin, hCG, and hMG for a total of 24 weeks. The control arm included 10 men who did not receive any hormonal stimulation. On repeat cTESE, 2/25 patients in the treatment group had successful sperm retrieval compared with 0/10 patients in the control group.

There is a paucity of controlled studies investigating the use of hormone stimulation therapy in both primary and secondary mTESE. Therefore, there is a need for large-scale prospective randomized controlled studies to elucidate the benefits of hormone stimulation therapy in men with NOA with a negative mTESE.

## Varicocele repair

The value of varicocele repair in the context of NOA remains debatable. Esteves et al.^41^ conducted a meta-analysis, which compared the surgical sperm retrieval and pregnancy rates after varicocele repair in NOA. The authors reported a trend toward a higher pregnancy rate in the varicocele repair group, but this was not statistically significant. However, there was a significantly increased surgical sperm retrieval rate associated with varicocele repair (odds ratio [OR]: 2.65, p<0.001). The impact of varicocele repair on live birth rate (OR: 2.19, p=0.05) was observed to be not statistically significant. However, this study was limited because the meta-analysis included only 3 controlled studies.

The meta-analysis by Kirby et al.^42^ reported a significantly improved surgical sperm retrieval rate (OR: 2.509, p=0.001) and clinical pregnancy rate (OR:2.34, p=0.044) after varicocele repair compared with the control group. The authors observed a non-significant increase in the live birth rate in the varicocele repair cohort compared with the control group. However, this meta-analysis included only 2 studies; therefore, its findings are weakened by the limited data set.

Weedin et al.^43^ assessed whether testicular histology affected the impact of varicocele repair in NOA. This meta-analysis showed that after varicocele repair, the histological subtypes of hypospermatogenesis (OR: 9.4, p<0.001) and maturation arrest (OR: 5.7, p<0.001) had a significantly higher probability of motile sperm production in the ejaculate or spontaneous pregnancy compared with SCO histology. However, no randomized controlled trials or prospective studies were included in this review. Moreover, the data included in this analysis did not contain a control group, and histopathological information, such as whether the final histopathology was defined by the most prominent or most favorable pattern seen, is not reported. Sönmez et al.^44^ noted that in 5%–35% of men with NOA, there is intermittent sperm production in the ejaculate, and this highlights the importance of a control group to discern the impact of varicocele repair on sperm production in NOA. Moreover, it has been reported that 55.5% of men with NOA who produce sperm in their ejaculate after varicocele repair will revert to azoospermia within 1 year of the procedure.^44^ Schlegel et al.^45^ reported that only 9.6% of men, after varicocele repair, would have sufficient viable sperm in the ejaculate to avoid a TESE. Moreover, Lee et al.^46^ performed an economic analysis of data from the society for assisted reproductive technology database, peer-reviewed literature, the medicare resource-based relative value scale, and sampling of high volume in vitro fertilization (IVF) centers in the United States. The authors reported that mTESE was a more cost-effective treatment than varicocele repair in the management of NOA infertility ($65,515 vs $76,878).

In summary, varicocele repair can improve semen parameters in NOA, but its impact on pregnancy or live birth rates is questionable. There is a paucity of randomized controlled trials, and the current literature is limited to retrospective data.

## Experimental Techniques

We reviewed promising technological advancements, which could play a vital role in optimizing surgical sperm retrieval surgery and prove to be effective in men with failed mTESE.

## Use of round spermatids for ART

In cases of mTESE that have failed to identify spermatozoa or elongated spermatids, round spermatids have been sampled and used in ART. Round spermatids are immature sperm that have not yet completed the maturation stage of spermiogenesis and hence not undergone processes, such as DNA condensation and acrosome and flagellum formation.^47^ Tanaka et al.^48^ observed that in a cohort of 730 men with NOA who had previously failed mTESE, a repeat mTESE identified round spermatids in 10.4% (76). Moreover, the use of round spermatids in 163 cycles of IVF resulted in the births of 14 healthy babies. However, owing to the low live birth rate, it is difficult to determine the safety of ART using round spermatids. The current literature shows that the use of round spermatids in IVF has limited success, and questions have been raised about the feasibility of accurately identifying round spermatids from diploid precursors.^49^ Furthermore, there are theoretical concerns regarding the potential adverse health issues in offspring conceived by round spermatids.^49^ Therefore, there is a need for randomized prospective controlled studies with a risk–benefit analysis.

## Stem cell therapy

Stem cell therapy remains an experimental treatment in the management of male infertility. Stem cells are derived from spermatogonial stem cells (SSCs), which are located near the basal lamina of seminiferous tubules and pluripotent stem cells (PSCs) from either embryonic origins or induced from somatic cell types.

SSCs are capable of self-renewal and differentiation into mature spermatozoa depending on the microenvironment, otherwise known as the stem cell niche.^50^ There are 2 methods of SSCs transplantation: autologous testicular tissue grafting and isolated SSCs injection. Fayomi et al.^51^ successfully reported the first live offspring of a non-human primate born from the sperm extracted from a scrotal graft. An advantage of testicular tissue grafting is the potential preservation of the natural SSCs niche. However, this would only apply in cases of NOA where the patient had previously functional SSCs, which were biopsied and preserved. An example of this would be, in childhood cancer survivors who had banked testicular tissue before commencing gonadotoxic therapies.

Isolation and *ex vivo* expansion of SSCs could potentially have more varied applications, such as repopulating the seminiferous tubules in SCO, activating dormant or suppressed cells in maturation arrest, and enhancing spermatogenesis in men with insufficient but functional SSCs.^52^ Long-term propagation of human SSCs has been achieved with cell culture techniques, which have been reported to confer stable genetic and epigenetic profiles.^53^ Goossens et al.^54^ reported no difference in DNA methylation patterns and fetal developments in 2 generations of murine offspring after conception via SSCs transplantation in genetically sterile male and fertile female mice. These successes have not been replicated in non-human primates, and this may be owing to the species-specific requirements of cell culture conditions and challenges in isolating SSCs from biopsy samples.^55^

PSCs have the ability to differentiate into different cell types. There are ethical restrictions from obtaining PSCs from the inner cell mass of an embryo but PSCs can be induced from somatic cells, such as dermal fibroblasts. Induced PSCs (iPSCs) have been successfully derived from a patient with Klinefelter’s syndrome.^56^ In murine models, iPSCs have been successfully developed into primordial germ cell-like cells *in vitro* and transplanted into the testes of mice leading to live births.^57,58^ Mice skin fibroblasts have been reprogrammed into embryonic Sertoli cells^59^ and Leydig-like cells,^60^ which were able to restore the testosterone levels *in vivo.*^60^ PSCs can theoretically be used to restore non-functional SSCs niches and support *ex vivo* SSCs expansion. However, studies in human iPSCs are still in its infancy because the induction and differentiation specifications are different in human iPSCs than murine iPSCs.^61,62^ Another significant limitation in using iPSCs is the accumulation of genetic and epigenetic mutations during reprogramming and expansion, which could result in unwanted germ-line mutations.

## Technology

There is ongoing research to identify whether the use of technology can optimize sperm retrieval surgery. Multiphoton microscopy (MM) allows for visualization of the seminiferous tubule cellular architecture.^63^ MM uses near-infrared lasers to cause fluorophores, such as nicotinamide adenine dinucleotide phosphate, to produce autofluorescence.^64,65^ This in conjunction with second harmonic generation results in real-time imaging of tissues.^64,65^ Ramasamy et al.^63^ reported that the use of MM on rodent testes allowed discrimination of seminiferous tubules, which contained sperm, from those that did not. Najari et al.^66^ correlated MM imaging of *ex vivo* testicular biopsies with the histological diagnosis from hematoxylin and eosin–stained tissue. The authors observed a concordance rate of 86% between MM imaging and histology. However, the cohort size was only 7 patients. Furthermore, before being used in clinical practice, the safety profile of MM has to be confirmed considering the potential adverse effects of lasers on the sperm, including DNA fragmentation.^66^

Full-field optical coherence tomography (FOCT) applies white-light interferometry to testicular tissue to provide detailed tomographic images.^67,68^ Ramasamy et al.^68^ applied FOCT on *ex vivo* testicular tissue biopsies taken from bulsafan-treated rats (to simulate SCO). The authors reported that FOCT was able to identify the seminiferous tubules undergoing spermatogenesis, and these findings correlated with histological hematoxylin- and eosin-stained images. However, the FOCT device used was only able to examine the testicular tissue *ex vivo*; thus, this technique can be used to confirm the presence of sperm in the testicular tissue samples rather than aid extraction. Moreover, FOCT was criticized because of its limited depth of imaging and inability to provide cellular details.^68^

Although these technologies suggest promising adjuncts to mTESE, they need to be evaluated in large-scale human studies before use in clinical practice.

## Predictive modeling

Testicular size and serum FSH levels have been purported to be predictors of sperm retrieval outcomes; however, the data in the literature are inconsistent.^7, 12, 69^ Predictive modeling and composite markers have been tested to provide a more accurate discriminatory ability. Ramasamy et al.^70^ applied artificial neural networks to develop algorithms to predict mTESE outcomes in men with NOA. The authors reported that the neural network was able to predict the outcome in 152/256 (59.4%) of the patients tested.

The use of predictive modeling represents an exciting prospect as it may allow the use of personalized medicine and provide the clinician with the necessary information to counsel the patients on the likelihood of successful sperm retrieval in repeat mTESE.

## Conclusion

**Figure 1.**
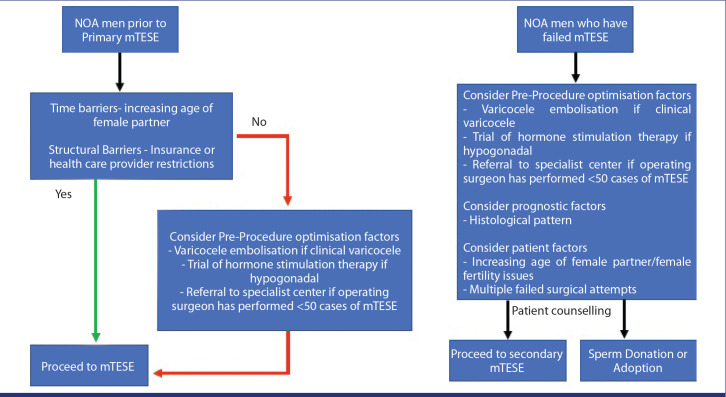
Algorithm for non-obstructive azoospermia and microdissection testicular sperm extraction

A recent meta-analysis reported that the sperm retrieval rate from mTESE was 46%.^7^ Therefore, counseling a patient regarding a failed mTESE is not uncommon. Unfortunately, there is a paucity of welldesigned, large-scale studies to guide the clinician on the management strategies in this scenario. We have designed an algorithm (Figure 1) on how to approach men with NOA who have never had an mTESE and those who have failed mTESE.

Although hormone stimulation therapy and FNA mapping may optimize sperm retrieval surgery, there are insufficient data to suggest that it may improve the outcomes in men who have failed mTESE. Moreover, given the specialist equipment and expertise required to perform mTESE, it is rare that an operating surgeon would not have performed the required 50 cases (the recommended learning curve) for optimal expertise. Hence, in the vast majority of failed mTESE cases the management strategies are limited and include adoption or sperm donation. A repeat mTESE can be attempted, but the authors recommend hormone stimulation therapy in patients with hypogonadism on the rationale that there is a theoretical plausibility that this may improve spermatogenesis. The advent of newer technologies, such as MPM, represents promising tools for identifying areas of focal spermatogenesis, but in the absence of human trials, these adjuncts are some way from entering clinical practice. Moreover, predictive modeling databases are still in their infancy but with more robust databases, it could provide a critical tool in patient counseling.

It is also important to appreciate both structural barriers and patient factors. For example, increasing female age (>35 years) is associated with poor ART outcomes; therefore, the time delay associated with hormone stimulation therapy, varicocele repair, or FNA mapping may not be advisable in older couples.^71^ Furthermore, many insurance or healthcare providers may not permit any additional treatments or set an age limit in couples receiving ART. Given that mTESE necessitates extracting testicular tissue, multiple attempts can theoretically increase the risk of testicular atrophy and subsequent hypogonadism.

Thus, couples should be counseled about all the options available, including sperm donation or adoption, and a joint decision should be made.

## Figures and Tables

**Table 1.** Sperm retrieval rates for the 6 largest microdissection testicular sperm extraction studies reported in a recent meta-analysis by Corona et al.^7^

Study	Sample Size	Successful Sperm Retrieval	Sperm Retrieval Rate (%)
Chehrazi et al.,^10^ 2017	537	119	22.1
Althakafi et al.,^11^ 2017	421	166	39.4
Bryson et al.,^12^ 2014	1127	631	56.0
Berookhim et al.,^13^ 2014	640	285	44.5
Karacan et al.,^14^ 2013	406	223	54.9
Ramasamy et al.,^15^ 2009	792	475	60.0
